# Titanium dioxide nanoparticles impart protection from ultraviolet irradiation to fermenting yeast cells

**DOI:** 10.1016/j.bbrep.2022.101221

**Published:** 2022-02-04

**Authors:** Yui Ono, Hitoshi Iwahashi

**Affiliations:** The Graduate School of Applied Biological Sciences, Gifu University, Tokai National Higher Education and Research System, Yanagido 1-1, Gifu City, Gifu prefecture, Japan

**Keywords:** Titanium dioxide, UV, Yeast, Reactive oxygen species, Oxidative, Reductive

## Abstract

The photocatalytic activity of titanium dioxide is widely utilized in science and technology. In the biological field, titanium dioxide is believed to be a disinfectant because it produces reactive oxygen species (ROS). However, there are multiple types of ROS such as hydroxyl radicals, superoxide anions, singlet oxygen, and hydrogen peroxide. In this study, we attempted to characterize the various mechanisms and roles of ROS in disinfection. Surprisingly, we found that titanium dioxide protected yeast cells from ultraviolet irradiation. We characterized the ROS produced under these conditions. The production of hydroxyl radicals and superoxide anions was confirmed; however, glucose in the yeast medium scavenged hydroxyl radicals. The photocatalytic activity of titanium dioxide produced oxidative products and reductive products, as oxidation and reduction occurred simultaneously. Once hydroxyl radicals are scavenged, the photocatalytic activity of titanium dioxide produces a reductive environment for fermenting yeast cells and protects them from oxidative stress by ultraviolet irradiation.

## Highlights

• Titanium oxide nanoparticles did produce hydroxyl radical and superoxide anion under UV irradiation.
• Glucose in medium play the role as scavenger to hydroxyl radical.
• Titanium oxide nanoparticles in medium apparently produce superoxide anion under UV.
• Titanium oxide nanoparticles in medium make their environment reduction state.
• The reduction state protects yeast cells from oxidative stress under UV.

## 1 Introduction

The photocatalytic activity of titanium dioxide [1] is widely accepted and reviewed [2,3], and this activity produces reactive oxygen species (ROS). These ROS are believed to oxidize a wide range of organic contaminants [4] as well as to destroy microbial cells [2]. However, oxidation and reduction occur together, that is, the give and take of electrons. The substrate of the photocatalytic activity of titanium dioxide is water molecules, and not contaminants or microbes, which can be damaged only by the photocatalytic reaction products.

The photocatalytic activity of titanium dioxide was discovered by Honda and Fujishima [1]. Light-irradiated titanium dioxide suspended in water produces free electrons and holes. Electron holes are created by the loss of electrons. The holes take electrons from water molecules and produce hydroxyl radicals. This is the principle of the Honda–Fujishima effect [1]. These reactions are oxidative and reductive reactions, and the products are oxidative and reductive products. Oxidation and reduction occur simultaneously, with no exceptions. Under aerobic conditions, the dissolved oxygen in water accepts electrons to form superoxide anions. The Honda-Fujishima effect can be monitored by the production of hydroxyl radicals and superoxide anions with titanium dioxide nanoparticles under submerged and UV or X-ray exposure conditions [5]. The photocatalytic production of ROS is not limited to metal-oxide particles. ROS production in cancer cells by protoporphyrin IX is accepted as a photodynamic therapy [6]. Recently, flavonoids of luteolin and quercetin have been characterized as photocatalytic natural chemicals [[7], [8]]; approximately 300 of 9600 natural chemicals have been listed as producers of ROS by X-ray irradiation [9]. These chemicals produce ROS of hydroxyl radicals and superoxide anions under irradiation.

Hydroxyl radicals and superoxide anions are well known as ROS and are believed to cause damage to organisms. Thus, there are many reports that show titanium dioxide toxicity, especially nanoparticles of titanium dioxide, under light irradiation [10], with evidence of oxidative stress responses. We previously tried to detect the toxicity of anatase titanium dioxide nanoparticles under UV irradiation using microbes of the yeast *Saccharomyces cerevisiae* [[11], [12]]. However, we could not detect the toxicity caused by ROS, but rather found oxidative stress caused by UV irradiation alone [12]. Surprisingly, we observed a protective effect of titanium dioxide nanoparticles in UV irradiation [11]. Yeast cells were cultured on plate medium with titanium dioxide and we irradiated them with UV light. Under conditions where yeast cells could not grow due to UV irradiation, yeast cells with titanium dioxide grew on titanium dioxide particles [11]. Thus, it seems that titanium dioxide produced a protective effect in yeast cells.

In this report, we studied the mechanism of protection for titanium dioxide nanoparticles under lethal UV irradiation and found that exposure to UV irradiation produced a reductive environment. It is true that titanium dioxide produces ROS of hydroxyl radicals and superoxide anions under UV irradiation; however, glucose scavenges hydroxyl radicals when used as a medium component. It is necessary to monitor the species of ROS precisely, because the description “ROS induce oxidative stress” is not always correct.

## 2 Materials and methods

### 2.1 Titanium dioxide

In this experiment, titanium dioxide of anatase, 7 nm, ST-01 (Ishiharasangyo Co. Ltd, Japan) was used.

### 2.2 Organisms and growing condition

The yeasts *Saccharomyces cerevisiae* BY4741 (*MATa his3Δ1 leu2Δ0 met15Δ0 ura3Δ0*) and *S. cerevisiae* BY4741 containing pPTR2-4K>R-GFP were used as indicator organisms. pPTR2-4K>R-GFP is a shuttle vector containing Ptr2 fused by GFP, and the fused proteins are localized in the cellular membrane [13]. Yeast cells were grown at 30 °C in synthetic defined (SD, DIFCO, Japan) medium (2% glucose, 6.7 g/L yeast nitrogen base, 20 mg/l l-leucine, 60 mg/l l-histidine, 20 mg/l l-methionine, 20 mg/L uracil). For UV irradiation, yeast cells were spread onto SD agar medium (1.5% agarose).

### 2.3 UV irradiation

The UV lamp FL-10 BL-310 (Kyokkoudennki, Tokyo, Japan) at 310–320 nm (supplementary data) was used for irradiation. Titanium dioxide nanoparticle powders were scattered on the plates after spreading the yeast cells. The plates were incubated at 30 °C with UV irradiation by placing the plate medium upside down. To detect ROS, 96-well microplates containing ROS indicators were UV irradiated.

### 2.4 ROS detection

ROS detection assays were performed in microplates, and signals were detected using a SoftMax Pro 7 microplate reader (Molecular Devices Japan, Japan). Redox potential was measured by pH, ORP sensor of AS 800 (As one, Osaka, Japan) using ORP sensor.

To detect hydroxyl radical generation, we employed 3′-(*p*-aminophenyl) fluorescein (APF). APF was purchased from Sekisui Medical Co., Ltd., Japan. In the APF assay, samples were mixed with APF to a final concentration of 5 μM in 9.6 mM phosphate buffer pH 7.4 (PB) containing an appropriate concentration of titanium dioxide. The mixtures were excited at 480 nm and fluorescence was detected at 520 nm. The production of hydroxyl radicals was confirmed by comparing the fluorescence of the sample and the sample with ethanol of hydroxyl radical scavenger. Thus, the decreased fluorescence by the scavenger corresponding to the production of hydroxyl radicals.

To detect superoxide anion generation, we employed dihydroethidium (DHE). DHE was purchased from Invitrogen, CA,USA. DHE was mixed with the samples to a final concentration of 50 μM in PB containing an appropriate concentration of titanium dioxide. The mixtures were excited at 485 nm, and fluorescence responses were detected at 610 nm. The production of superoxide anion was confirmed by comparing the fluorescence of the sample and the sample with superoxide dismutase (SOD) as scavenger. Thus, the decreased fluorescence by the scavenger corresponding to the production of superoxide anion.

## 3 Results

### 3.1 Yeast cells create colonies when grown with titanium dioxide nanoparticles under UV irradiation

**Fig. 1:**
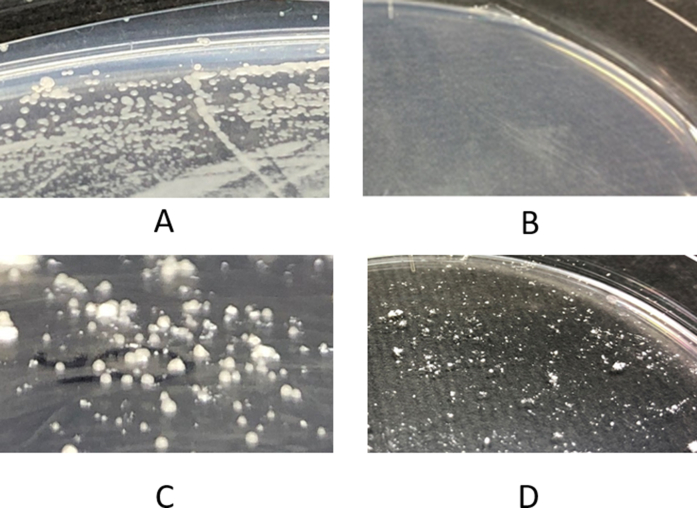
Colony formation of yeast cells with TiO_2_ under a lethal dose of 35 μW/cm³ UV irradiation. A, Yeast cells on SD medium; B, yeast cells on SD medium under UV irradiation; C, yeast cells on SD medium under UV irradiation and with TiO_2_; and D, TiO_2_ scattered on SD medium.

Fig. 1 shows the protective effect of titanium dioxide on yeast cells under UV irradiation at 35 μW/cm³. Yeast cells were spread on SD agar medium, titanium dioxide nanoparticle powders were scattered on the plates, and the plates were incubated at 30 °C (Fig. 1A). The plates were exposed to UV radiation from below. Under UV irradiation, yeast cells generally cannot form colonies (Fig. 1B); however, yeast cells formed colonies if the titanium dioxide nanoparticle powder was present (Fig. 1C). These titanium dioxide nanoparticles were sterilized, and no colonies were produced by the spread of titanium dioxide on the plate medium (Fig. 1D). We thus confirmed the protective effect of titanium dioxide against UV irradiation. The number of colonies was much lower than in plates not subjected to UV irradiation, but the colonies that formed did so in close proximity to the titanium dioxide particles.

### 3.2 Production of superoxide anions

**Fig. 2:**
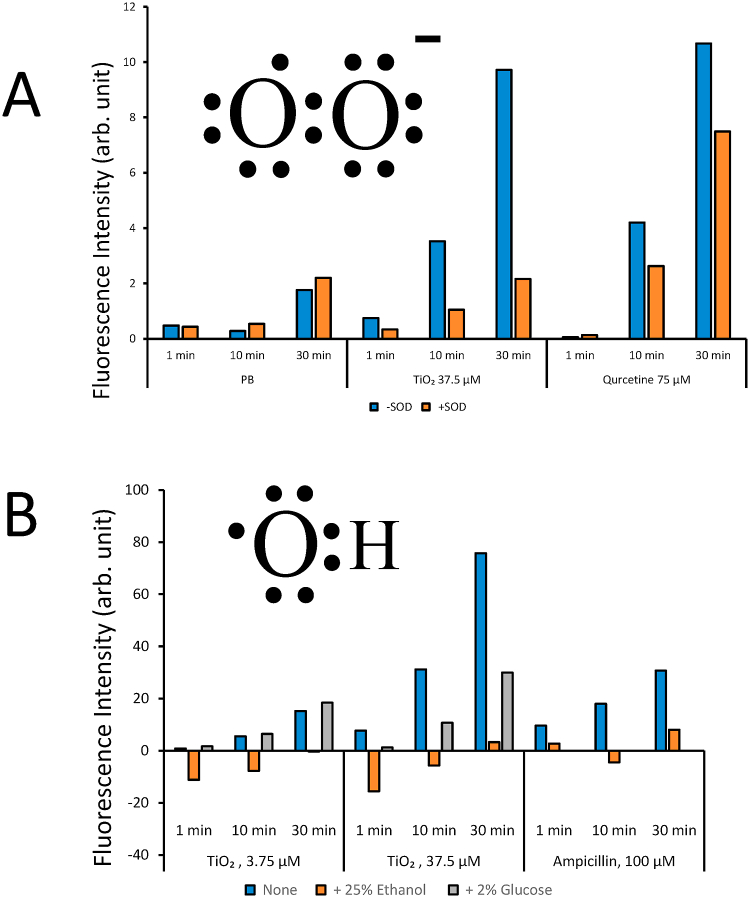
Production and scavenging of ROS under UV irradiation. A. Estimation of superoxide anion production and scavenging under 300 μW/cm³ UV irradiation. B, Production and scavenging of hydroxyl radicals by TiO_2_ suspensions under UV irradiation.

Many reports have shown that titanium dioxide produces ROS; however, ROS production is not always characterized. We estimated the production of superoxide and hydroxyl radicals. Fig. 2A shows the estimation of superoxide anion production under UV irradiation at 300 μW/cm³. Dihydroethidium (DHE) dissolved in DMF (Dimethylformamide) was diluted to a final concentration of 50 μM using PB. Ethidium production was monitored by excitation at 610 nm and emission at 485 nm, as shown by arbitrary units. DHE is a fluorescent probe that is reactive to superoxide anions; however, ethidium cation is an oxidative product of DHE. Superoxide anions are known to reduce cytochrome C [14]. DHE reactions with superoxide anions are not simple redox reactions, and ethanol production has been reported [15]. Thus, superoxide dismutase should be used as a specific scavenger for superoxide anions [16]. We found that 7.5 μM titanium dioxide suspension in PB showed approximately 10 arbitrary units without SOD and approximately 2 arbitrary units with SOD after 30 min of UV irradiation; thus, titanium dioxide produced superoxide anions at a level of 8 arbitrary units under our experimental conditions. This production was higher than that obtained with 75 μM quercetin. Quercetin is a superoxide-producing natural chemical when exposed to UV and X-ray radiation [8]. We detected the production of superoxide anions after UV exposure of titanium dioxide suspended in PB.

### 3.3 Production of hydroxyl radical

Fig. 2B illustrates the production of hydroxyl radicals by titanium dioxide suspensions under UV irradiation with 300 μW/cm³. Aminophenyl fluorescein (APF) with ethanol was employed for estimation of hydroxyl radical, as ethanol has been shown to be a scavenger to hydroxyl radicals [5]. APF is oxidized by hydroxyl radicals and fluorescein is produced. Fluorescein is a strong fluorescence chemical and can be monitored with 520 nm excitation and 490 nm fluorescence. Thirty minutes of UV irradiation in a 37.5 μM titanium dioxide suspension showed significantly high fluorescence, and this fluorescence was scavenged by ethanol. Ampicillin was used as a positive control for hydroxyl radical production [9]. We could also detect the production of hydroxy radicals after UV exposure of titanium dioxide suspended in PB.

### 3.4 Glucose as scavenger of hydroxyl radicals

We confirmed the protective effect of titanium dioxide against UV irradiation (Fig. 1). We hypothesized that the titanium dioxide particles were inducing the production an unknown substance which protected the yeast cells from UV irradiation. We previously suggested that UV irradiation causes oxidative stress in yeast cells under these conditions [12]. Yeast cells may overcome this oxidative stress by means of the generation of yet uncharacterized substances when in contact with titanium dioxide, which may have reductive effects on fermenting yeast cells. We confirmed that titanium dioxide produced hydroxyl radicals and superoxide anions under UV irradiation. The hydroxyl radical is an oxidant, as it requires electrons to become stable hydroxide anions (Fig. 2B), whereas the superoxide anion is a reductant because it is electron-rich (Fig. 2A). Superoxide anions are known to reduce cytochrome C and can be used as reductants in yeast cells. How can titanium dioxide selectively produce a reductant? It is known that hydroxyl radicals are scavenged by some chemicals; it is possible that such chemicals exist in the medium or metabolites of yeast cells. We speculated that glucose or agarose could scavenge hydroxyl radicals. These chemicals have several hydroxyl groups, which can scavenge hydroxyl radicals such as ethanol and flavonoids [8]. If the glucose in the medium could scavenge hydroxyl radicals, titanium dioxide could produce electrons or superoxide anions under UV irradiation. To confirm this speculation, we evaluated the ability of glucose to act as a hydroxyl radical scavenger. Fig. 2B shows the scavenging ability of the 2% glucose solution. The addition of glucose clearly decreased the fluorescence intensity of fluorescein.

### 3.5 Reduction of oxidized cytochrome C by titanium dioxide under UV irradiation in the presence of glucose

**Fig. 3:**
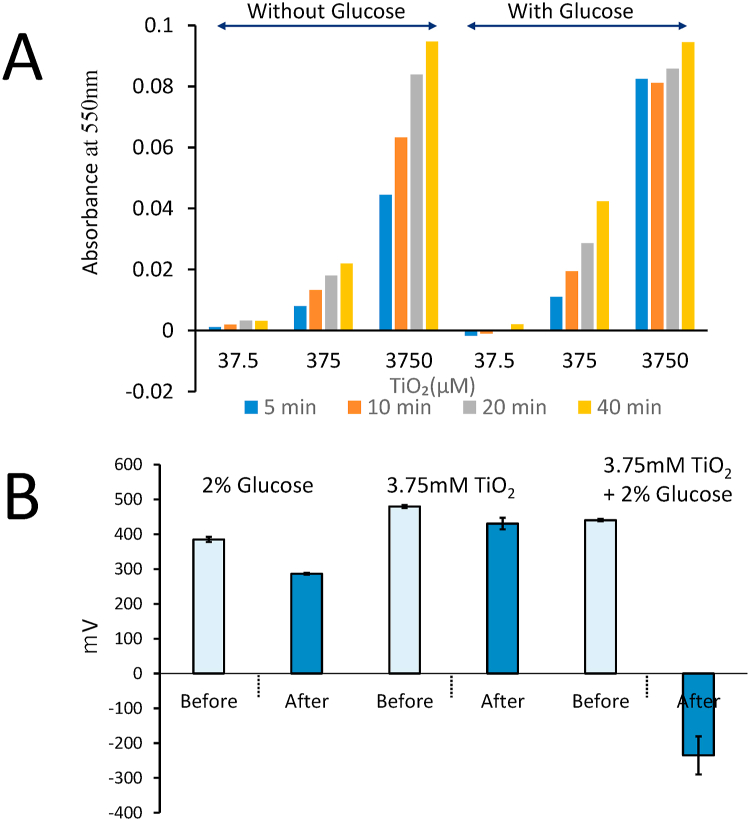
Reducing ability of TiO_2_ under UV irradiation in the presence of glucose. A, Reduction of oxidized cytochrome C to reduce cytochrome C under UV irradiation. B, Redox potential of TiO_2_ suspensions in a 2% glucose solution under UV irradiation.

Glucose scavenges hydroxyl radicals; and this suggests that titanium dioxide produces electrons and has a reducing effect under UV irradiation. We estimated the reducing ability of titanium dioxide under UV irradiation with glucose. The reducing ability was monitored by measuring the reduction of oxidized cytochrome C to reduced cytochrome C. The color of oxidized cytochrome C is dark and becomes light red when reduced; reduced cytochrome C has a specific absorbance at 550 nm. Fig. 3A illustrates the production of reduced cytochrome C from oxidized cytochrome C. The titanium dioxide suspension in PB reduced cytochrome C dependent on the amount of titanium oxide, and the medium with glucose reduced oxidized cytochrome C faster than that without glucose. This reduction is caused by electrons or superoxide anions produced under UV irradiation. We thus confirmed the reduction ability of titanium dioxide in glucose solution under UV irradiation.

### 3.6 Titanium dioxide produces a reductive environment with glucose under UV irradiation

To confirm that titanium dioxide in the glucose suspension produces a reductive environment, we measured the redox potential of titanium dioxide under glucose and UV combination conditions. We measured the potential under anaerobic conditions. Titanium dioxide suspensions (3.75 mM) were stirred to produce homogeneous irradiation. Before irradiation, the redox potential of the mixture was 440.3 mV; after irradiation for 20 h, that of the complete mixture of titanium dioxide, glucose, and UV irradiation was −235.1 mV. Before irradiation, the mixture without titanium dioxide and that without glucose generated 385.1 and 286.5 mA of current, respectively; after irradiation for 20 h or more, the redox potential of the mixtures measured 286.5 and 430.5 mV, respectively. (Fig. 3B). These results clearly show that titanium dioxide exposed to UV irradiation produces a reductive environment with glucose.

### 3.7 Yeast cells grow on the surface of titanium dioxide powder

**Fig. 4:**
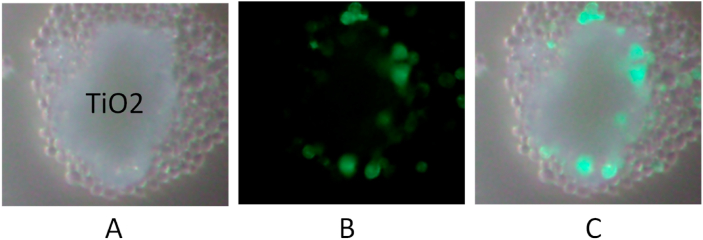
Living yeast cells around TiO_2_ particle surface. *Saccharomyces cerevisiae* BY4741 containing pPTR2-4K>R-GFP, which contains GFP-tagged Ptr2 proteins, was exposed to UV irradiation in the presence of TiO2 particles. A; microscopic image, B; fluorescence microscopic image, C; Merged image.

It is possible that aggregated titanium dioxide nanoparticles adsorbed yeast cells and provided cover from UV irradiation. We observed yeast cells that had GFP-labeled proteins localized on the cellular membrane. We expected the dead cells to have no GFP in the cellular membrane structure, unlike living cells. Fig. 4 shows the yeast cells around the titanium dioxide particles. We observed living yeast cells around the surface of the particles, which were not biased in one direction. (Fig. 4C). The cells separated from titanium dioxide had no GFP fluorescence and yeast could grow on the surface of titanium dioxide, but budded cells could be dead depending on the distance from titanium dioxide. We observed the growth of yeast cells for three days and we could not observe any cracks in the layer of titanium dioxide. If only the cells covered by titanium dioxide could grow, growing yeast cells inside the powders would not produce colonies without breaking the titanium dioxide powders. The fermenting yeast is surrounded by glucose, and we therefore conclude that titanium dioxide exposed to UV irradiation produces a reductive environment for fermenting yeast cells.

## 4 Discussion

In this report, we suggest that yeast cells could grow under UV irradiation when titanium dioxide was present. However, the evidences listed are limited to qualitative results. The indicators we employed have their own sensitivity. The most sensitive indicator was the yeast cells themselves, and 35 μW/cm³ of UV irradiation was sufficient to observe growth inhibition. The ROS detectors APF, DHE, and cytochrome-C are not very sensitive, and we observed the production of hydroxyl radicals, superoxide anions, and cytochrome-C reduction under 300 μW/cm³. Cytochrome C reduction requires titanium dioxide at 375 mg/mL under these conditions. To estimate the redox potential, we need more than 20 h. Thus, further experiments are needed to supplement descriptive studies and characterize the quantitative roles of ROS and the molecular types produced.

Even under the qualitative results, we may speculate the protection mechanisms for fermenting yeast cells to titanium dioxide nanoparticles under UV irradiation. We found titanium dioxide produce hydroxyl radicals and superoxide anions, and hydroxyl radicals are scavenged by glucose of medium component. The titanium oxide in yeast medium dominantly produces electrons and superoxide anions under UV irradiation. Moriyama et al. [9] reported the effects of titanium dioxide nanoparticle on yeast cells under UV irradiation. They suggested that yeast cells that are adsorbed by titanium dioxide under UV irradiation suffer oxidative stress using DNA microarray analysis. They did not show what function was damaged by oxidative stress. While, Bisquert et al. [17] showed antioxidant of melatonin protect yeast cells from UV irradiation. The protection was observed by lowering mortality and improving growth rate after exposure to UV. Antioxidants of natural compounds were reviewed to protect against the damaging effects of UV irradiation [18]. The reductive environment made by the combination of titanium dioxide, UV, and glucose can be the similar effect of antioxidants. This environment can be the reason to decrease oxidative stress caused by titanium UV irradiation. The functional characterization of protection mechanisms will be next target of those studies.

Many studies have shown the photocatalytic inactivation of microbes by titanium dioxide under light irradiation. In these experiments, microbes are suspended in a water-based solution, and microbes have no support from glucose or alcohols. Therefore, the microbes may die from exposure to ROS produced by photocatalytic activities; however, few publications have confirmed which ROS play the primary role in disinfecting. Details on the underlying mechanism and specific roles play by ROS in disinfection have not been published.

Many photocatalytic chemicals have been identified, including titanium dioxide, cerium dioxide, protoporphyrin IX, and 300 natural chemicals. These chemicals have been shown to produce ROS and are believed to play anti-cancer and sterilization roles, as well as exhibiting toxicity [2,[3], [5],]. It is known that ROS damage cells, microbes, and viruses. However, there is often an elementary misunderstanding that ROS is always an oxidant. “ROS” is often followed by “oxidative” without evidence at the molecular level. This combination of “ROS” and “oxidative” easily leads to the toxicity of materials or benefits for cancer therapy in the field of nanotoxicology and nanomedicine. Once the Lewis structure of the hydroxyl radical and superoxide anion (Fig. 2A and B) are observed, it is possible to identify them as oxidative molecules or reductive molecules, respectively.

Here, we suggest that ROS must be characterized by molecular type when discussing their benefits or disadvantages. At minimum, the “ROS” must be identified as a hydroxyl radical, superoxide anion or others.

## Funding information

This research was carried out by the Gifu University's own budget and no support from out side of University.

## Declaration of competing interest

The authors have no conflicts of interest directly relevant to the content of this article.

## Data Availability

Data will be made available on request.
