# Supplementary figures and images for: Titanium dioxide nanoparticles impart protection from ultraviolet irradiation to fermenting yeast cells
Source: Biochem Biophys Rep. 2022 Feb 4;30:101221. doi: 10.1016/j.bbrep.2022.101221 (PMC9171698; doi:10.1016/j.bbrep.2022.101221)

# FL-10 BL-310 分光スペクトル

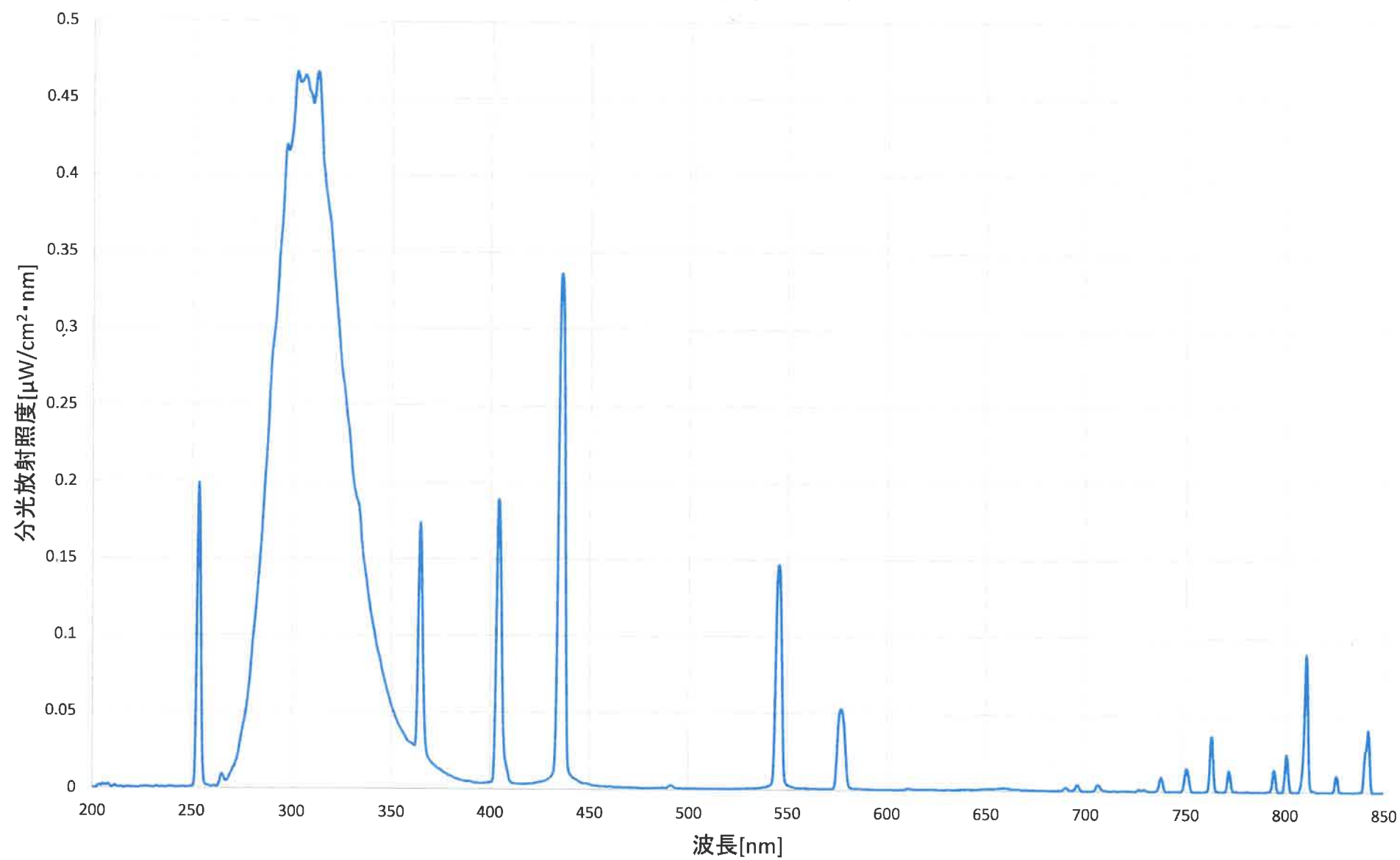

Supplement: Multimedia component 1 [file mmc1.pdf]
